# A261 SEVERE ACUTE LIVER INJURY SECONDARY TO WILSON’S DISEASE: A CASE REPORT AND REVIEW OF THE LITERATURE

**DOI:** 10.1093/jcag/gwac036.261

**Published:** 2023-03-07

**Authors:** J Besney, A Frolkis, J McLellan, S Congly, H Nguyen

**Affiliations:** 1 Division of Gastroenterology and Hepatology; 2 Department of Medicine; 3 Liver Unit, University of Calgary, Calgary, Canada

## Abstract

**Background:**

Wilson’s disease is autosomal recessive and rare (prevalence 1/30000-1/50000) caused by mutations in the *ATP7B* gene involved in copper excretion. Copper deposition drives hepatic injury, along with neuropsychiatric-ophthalmologic, and hematologic symptoms. Leipzig criteria greater than 4 establishes the diagnosis.

**Purpose:**

We report a case of severe liver injury secondary to probable Wilson’s disease.

**Method:**

Literature review of diagnosis, management including emerging therapies was performed using PubMed.

**Result(s):**

A 29-year-old otherwise healthy male with no family history of liver disease presented with abdominal pain, jaundice, and severe acute liver injury (ALT 1534 U/L ALP 114 U/L bilirubin 113 umol/L) initially misdiagnosed as hepatitis A in community urgent care. He had worsening liver injury (ALT 1809 U/L bilirubin 411 umol/L), with normal INR and no encephalopathy over an additional 3 weeks and was admitted to our tertiary care centre for further work up. History did not reveal viral prodromes, herbals or natural remedies. CBD oil was the only potential exposure, though longstanding use of the same brand argued against this as a cause. Chronic liver disease workup was negative (viral hepatitides, SARS-CoV-2, alpha-1-antitrypsin, acetaminophen level, iron profile, and comprehensive Mitogen autoantibody panel). Patent vasculature on Doppler US. Liver biopsy showed diffuse lobular inflammation, hepatocyte necrosis, 30% parenchymal involvement, primarily lymphocytes with some eosinophils and plasma cells, and no viral inclusions suggesting acute hepatitis with drugs or viral hepatitis as etiologies. The 24-hour urine copper was elevated at 2.19 umol/d (<0.80). Biopsy copper dry weight 60mcg/g (N<50; >250 diagnostic). Normal ceruloplasmin 0.30g/L and serum copper 25.8 umol/L. MRI brain not suggestive of Wilson’s disease, no Kayser-Fleischer rings on Ophthalmology assessment, and no hemolysis. Given a Leipzig score of 3 suggesting possible Wilson’s disease he was started on penicillamine 1500mg daily. Five weeks later he had serologic (ALT 1061 U/L bilirubin 128 umol/L) improvement, yet elevated elastography at 22kPa IQR 5% likely due to inflammation. Given coverage issues, he was switched to compassionate trientine 500mg BID with further improvement (ALT 347 U/L bilirubin 26 umol/L). Sequencing for *ATP7B* gene mutations are pending (Blueprint Genetics).

**Conclusion(s):**

Wilson’s disease should be considered in those with hepatic or neuropsychiatric symptoms, transaminases in the 1000s or acute liver failure. Treatment is lifelong with typically good prognosis and is fatal if untreated. Treatment options include chelators D-penicillamine (first line, with side effects including proteinuria and worsened neurological symptoms) and trientine (fewer side effects). Zinc prevents copper absorption and can be used in asymptomatic patients. Transplant in acute liver failure and end-stage liver disease is guided by the King’s score. Several novel therapies including gene therapy are in development.

**Please acknowledge all funding agencies by checking the applicable boxes below:**

None

**Disclosure of Interest:**

None Declared

